# Natural Selection on Exonic SNPs Shapes Allelic Expression Imbalance (AEI) Adaptability in Lung Cancer Progression

**DOI:** 10.3389/fgene.2020.00665

**Published:** 2020-06-24

**Authors:** Jinfei Huang, Yuchao Zhang, Qingyang Ma, Yuhang Zhang, Meng Wang, You Zhou, Zhihao Xing, Meiling Jin, Landian Hu, Xiangyin Kong

**Affiliations:** CAS Key Laboratory of Tissue Microenvironment and Tumor, Shanghai Institute of Nutrition and Health, University of Chinese Academy of Sciences, Chinese Academy of Sciences, Shanghai, China

**Keywords:** nature selection, allelic expression imbalance (AEI), expression noise, tumorigenesis, germline variants

## Abstract

Tumors are driven by a sequence of genetic and epigenetic alterations. Previous studies have mostly focused on the roles of somatic mutations in tumorigenesis, but how germline variants act is largely unknown. In this study, we hypothesized that allelic expression imbalance (AEI) participated in the process of germline variants on tumorigenesis. We screened single-nucleotide polymorphisms (SNPs) as representative germline variants. By using 127 patients’ RNA sequencing data from paired lung cancer and adjacent normal tissues from public databases, we analyzed the effects of the functional consequence of SNPs, function and conservativeness on genes with AEI. We found that natural selection can affect AEI. Functional adaptability of genes with a high frequency of AEI and a correlation of the incidence of AEI with conservativeness were observed in both adjacent tissues and tumor tissues. Moreover, we observed a higher incidence of AEI in genes with non-synonymous SNPs than in those with synonymous SNPs. However, we also found that AEI was affected by allele expression noise, especially in tumor tissues, which led to an increased proportion of AEI, weakened the effect of natural selection and eliminated the influence of the functional consequence of SNPs on AEI. We unveiled a previously unknown adaptive regulatory mechanism in which the effect of natural selection on SNPs can be reflected in allelic expression, which provides insight into a better understanding of cancer evolution.

## Introduction

Tumorigenesis is a process in which normal cells transform into cancer cells, manifesting as a cellular accumulation of changes at the genetic and epigenetic levels and eventually leading to uncontrolled proliferation. This process is similar to evolution, which acts through mutation accumulation and then selection (Nowell, 1976). A small number of cells with growth advantages obtain more clones and accumulate mutations until they transform into cancer cells. Previous studies have mostly focused on the roles of somatic mutations in tumorigenesis, but how germline variants act is largely unknown.

AEI refers to the phenomenon that the two alleles of genes exhibit unbalanced expression (Reinius and Sandberg, 2015). Previous studies have shown that expression quantitative trait loci (eQTLs) (Pickrell et al., 2010) and epigenetics (Shoemaker et al., 2010) can cause AEI. Indeed, *APC* (Pavicic et al., 2014), *CDH1* (Pinheiro et al., 2010), *CAT* (Yeo et al., 2014), and *TGFBR1* (Valle et al., 2008) have demonstrated higher percentages of AEI in cancer. Knudson proposed that people who carried heterozygous germline mutation of tumor suppressor genes were more likely to develop cancer than those who did not (Knudson, 1971). We hypothesized that in the absence of new mutations as “the second hit,” allele dosage changes can directly reflect the effects of heterozygous SNPs.

In this study, we screened heterozygous SNPs and carried out a genome-wide analysis on AEI in lung adenocarcinoma, and we systematically investigated the characteristics of natural selection at the allele expression level. We unveiled a previously unknown regulatory role in which the effect of natural selection on SNPs can be reflected in allelic expression. Some characteristics of natural selection at the level of gene expression through AEI are similar to how natural selection works on the genome. On the other hand, tumor tissues have a higher incidence of AEI than normal tissues, but the effect of expression noise on a single allele attenuates the effect of natural selection. We speculate that differences in AEI patterns between normal tissues and tumor tissues underscore the contribution of common SNPs to cancer progression.

## Results

### Tumor Samples Have a Higher Proportion of AEI Than Adjacent Tissue Samples

We systematically analyzed DNA and RNA sequencing data from lung adenocarcinoma–normal lung tissue pairs from The Cancer Genome Atlas (TCGA), and also analyzed RNA sequencing data from a South Korean dataset (Seo et al., 2012) for verification. We called variants from RNA-seq data for every paired cancer-normal sample, and we selected the heterozygous variants as candidate sites (see section “Materials and Methods”). To reduce the false rate caused by systematic mismatches of next-generation sequencing and to reduce the effect of somatic mutation and RNA editing, we downloaded the annotation file from the ftp servers of dbSNP (dbSNPBuildID=142) (Sherry et al., 2001) and retained the SNPs from our analysis that overlapped with the dbSNP. We found 40456 heterozygous SNPs in 11733 genes in TCGA data and 41096 heterozygous SNPs in 11846 genes in South Korean data. The probability density distribution showed that most genes (87.5% in adjacent samples and 85.4% in tumor samples) had average heterozygous SNPs of less than 1.5 in TCGA data (Supplementary Figure S1). We used the ratio of reads that covered the heterozygous SNPs to represent the allele ratio of AEI genes. Moreover, instead of setting a fixed cut-off of the expression ratio of two alleles, we used a probability distribution to define AEI to reduce false positives in genes with low expression.

**FIGURE 1:**
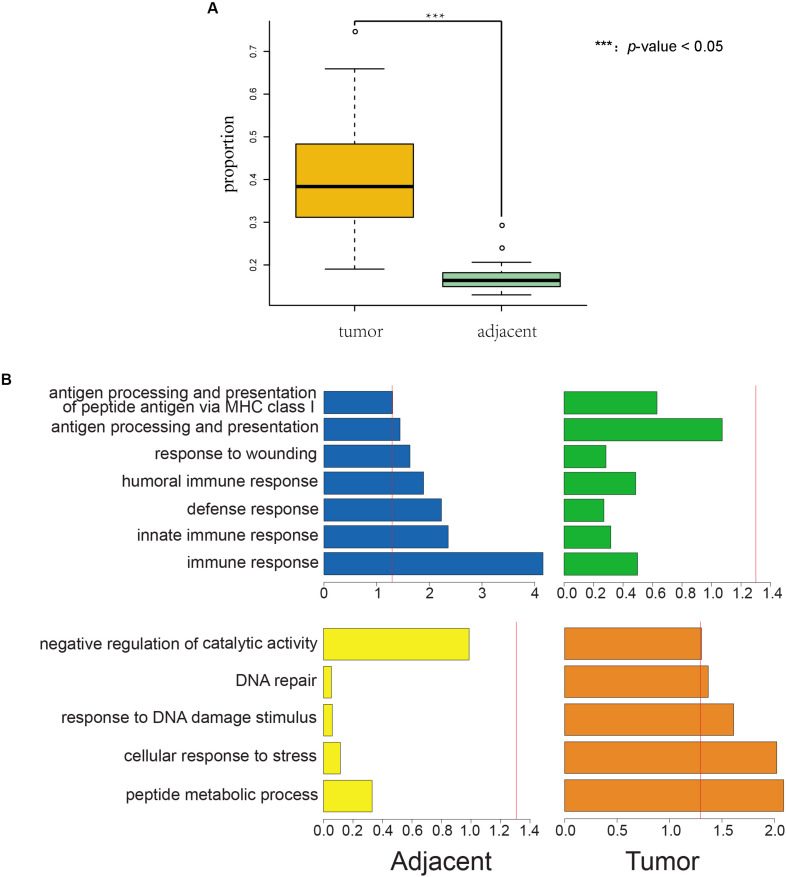
Proportions of AEI in tumor samples are significantly higher than those in adjacent samples, and AEI genes in cancer and adjacent tissues conform to organizational function. **(A)** A *t*-test of proportions of AEI in adjacent (green) and cancer (yellow) samples from RNA-seq data shows proportions of AEI in tumors are significantly higher (*p-*value = 1.68 × 10^– 37^) than those in adjacent samples from TCGA data. **(B)** Gene function cluster analysis for commonly expressed genes that have different frequencies of AEI in tumor and adjacent tissues. The *p*-value of the clustering term was taken as a negative logarithm. (blue and yellow) Gene function cluster analysis for genes that have high proportions of AEI in adjacent samples and low proportions of AEI in tumor samples. (green and orange) Gene function cluster analysis for genes that have high proportions of AEI in tumor samples and low proportions of AEI in adjacent samples. The red line represents a *p-*value = 0.05.

We found that the incidence of AEI was significantly higher in cancer samples than in adjacent samples for both TCGA (*p-*value = 1.68E^–37^, Figure 1A, and Supplementary Table S1) and South Korean samples (*p*-value = 5.55E^–14^, Supplementary Figure S2 and Supplementary Table S2).

As reported, somatic variation, allelic loss (Jen et al., 1994), or allelic copy number changes (Zack et al., 2013) affect allele bias at the chromosome level. In addition, changes in copy number in tumor genomes are associated with AEI (Tuch et al., 2010). To observe the effects of allele bias on AEI, we analyzed exome sequencing and whole-genome sequencing data from TCGA samples. The proportion of allele bias in tumors was higher than that in adjacent samples (*p-*value = 0.0282) (Supplementary Figure S3A). Notably, after removing genes with allele bias, we still observed that the proportion of AEI in tumor samples was significantly higher than that in adjacent tissues (*p*-value = 1.06*E*^−8^) (Supplementary Figure S3B).

### Tumor-Specific and Normal-Specific AEI Genes Show Different Functional Enrichment

Evolution means a change in the allele frequency, even a loss of the allele (Takeda and Matsuoka, 2008). AEI is equivalent to a change in allele frequency at the expression level. Previous AEI studies for tumor-associated genes also showed that allelic dose changes were consistent with the direction of survival of the appropriate tumor, which was consistent with the results of natural selection (Valle et al., 2008; Pinheiro et al., 2010; Pavicic et al., 2014; Yeo et al., 2014). There were also studies showing that RNA editing is generally non-adaptive (Xu and Zhang, 2014). This evidence suggests that natural selection will be reflected at the transcriptome level.

We hypothesized that AEI could be subject to natural selection. We defined the frequency of AEI as the percentage of individuals who had AEI in the specific genes, and we considered only genes that were expressed in at least 20% of patients. The frequencies of AEI are displayed in Supplementary Tables S3, S4. The probability density distributions of the frequencies of AEI are shown (Supplementary Figure S4), and we selected the fourth quartile of the frequency as the cut-off for high frequencies of AEI. To study the functional relevance of genes with different frequencies of AEI between tumor and adjacent samples, we screened non-specifically expressed genes in tumor and adjacent samples as background, genes that had high frequencies of AEI in the tumor samples (tumor group), and genes that had high frequencies of AEI in the adjacent samples (adjacent group).

We used DAVID (Database for Annotation, Visualization and Integrated Discovery) (Dennis et al., 2003) to study the enrichment of biological processes (BPs) in tumor and adjacent groups (details in section “Materials and Methods”). We found that genes in the adjacent group were enriched in immune-related annotations but in the tumor group were not (Figure 1B blue and green).

In addition, genes in the tumor group were enriched in stress reactions and DNA repair but in the adjacent group were not (Figure 1B yellow and orange).

Genes with a high frequency of AEI in tumor and normal tissues showed different tendencies of function. We also observed the result of enrichment was less obvious in tumor tissues than in adjacent tissues.

### Conservative Sites Had Less Frequent AEI in Normal Tissues but Higher AEI in Cancer Tissues

**FIGURE 2:**
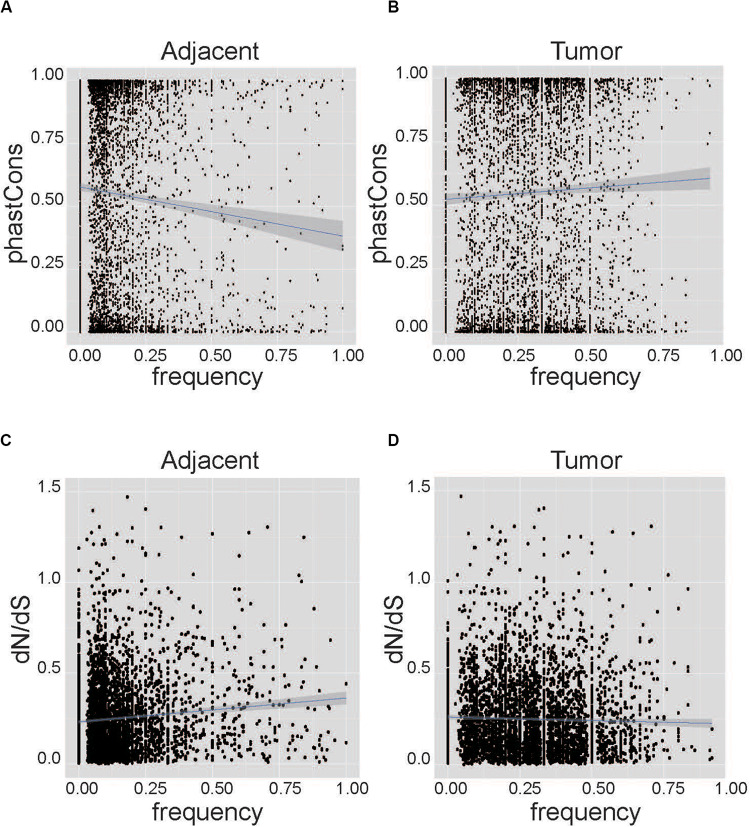
Correlation analysis between conservation and the frequency of AEI at the nucleotide level. The horizontal axis represents the frequency of AEI in the gene in the patient sample, and the vertical axis represents conservation using the PhastCons score and dN/dS. **(A)** The correlation analysis for adjacent tissues shows that sites with higher conservation scores tend to have lower frequencies of AEI (Cor = –0.08, *p-*value = 1.97*E*^−8^). **(B)** The correlation analysis for cancer tissues shows that more conservative sites tend to have higher frequencies of AEI (Cor = 0.04, *p-*value = 0.00604). **(C)** The correlation analysis for adjacent tissues shows that genes with higher frequencies of AEI also have higher dN/dS (Cor = 0.11, *p*-value = 4.34*E*^−13^). **(D)** A correlation between dN/dS and the frequency of AEI in tumor tissues could not be observed (Cor = –0.02, *p-*value = 0.19).

We performed correlation analysis between conservation and the frequency of AEI at the nucleotide level and the gene level. At the nucleotide level, PhastCons conservation data from UCSC can represent the possibility of each nucleotide being conservative (Sadri et al., 2011; Karolchik et al., 2014). The sites with higher PhastCons scores tended to have low frequencies of AEI in adjacent samples (Cor = −0.08, *p*-value = 1.97E^–8^, Figure 2A). Conversely, in tumor samples, SNPs with higher PhastCons scores tended to have higher frequencies of AEI (Cor = 0.04, *p*-value = 0.00604, Figure 2B, and Supplementary Table S3). We repeated the analysis on the South Korean data and found the same results (Supplementary Figure S5 and Supplementary Table S4).

At the gene level, the ratio of the non-synonymous nucleotide substitution rate to the synonymous rate (dN/dS) was used to represent conservation at the gene level (Miyata and Yasunaga, 1980). Genes with high frequencies of AEI tended to have high dN/dS in adjacent tissues (Cor = 0.11, *p-*value = 4.34E^–13^, Figure 2C); however, in tumor tissues, this tendency was not apparent (Cor = −0.02, *p-*value = 0.19, Figure 2D, and Supplementary Table S3). The same result was found in the South Korean data (Supplementary Figure S6 and Supplementary Table S4).

Taken together, we found a significant correlation between conservation and the frequency of AEI at both the nucleotide level and the gene level in adjacent samples but not in tumor samples.

### The Expression Ratio of Two Alleles Is More Random in Tumor Tissue

From the above analyses, we know that AEI occurs more frequently in tumor samples, which seems to indicate that tumors are affected more strongly by natural selection than normal tissue, but the decreased correlation between AEI and gene function, as well as conservation, indicates that tumors are less affected. If only natural selection is used to explain these results, there will be conflicts. We hypothesized that other factors must also affect AEI.

Even if two cells have the same genotype and external environment, the two cells can still have different phenotypes, and this phenomenon is called gene expression noise (Raj and van Oudenaarden, 2008). Compared to normal tissues, expression noise in tumor tissues is increased (Han et al., 2016). Thus, we inferred that expression noise could be applied to AEI and that the high incidence of AEI in tumors could be caused by noise rather than by natural selection.

**FIGURE 3:**
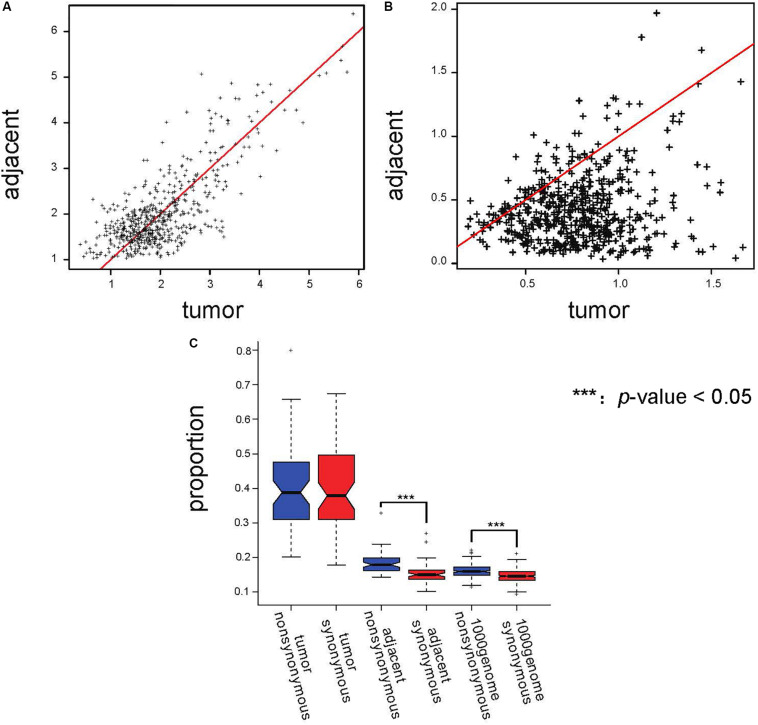
Proportions of AEI in tumor samples did not show a stronger selection pressure; instead, they showed a stronger stochastic process. **(A)** Scatterplot of the average major/minor for the adjacent and cancer samples. The red line represents x = y. **(B)** Scatterplot of the major/minor coefficients of variation (CV) for the adjacent and cancer samples. The red line represents x = y. **(C)** A *t*-test analyzing the proportions of AEI in all expressed genes with synonymous (red) and non-synonymous (blue) SNPs in tumor samples, adjacent samples and 1000 Genomes data. In the tumor samples, no significant promotion of non-synonymous SNPs was observed.

We screened the AEI genes in adjacent tissues and calculated the expression ratio of the two alleles. We defined the allele that had a higher allele expression ratio in the majority of adjacent tissues as the major allele and the other as the minor allele. Then, we calculated the mean and the coefficient of variation (CV) of the ratio of the major allele to the minor allele (major/minor) for every AEI gene expressed in at least 10 paired samples from TCGA data. Because CV represents the degree of heterogeneity of the allele expression ratio of the genes in different samples, we used CV as a measure of noise. The scatterplot of the mean major/minor (Figure 3A) showed that there was no obvious difference between the ratio in tumor samples and the ratio in adjacent samples. Paired *t*-test also suggested the there was no significant difference between the ratio in tumor samples and in adjacent samples (*p*-value = 0.401). The results demonstrated that the allele ratio did not significantly change in the tumor, and tumor tissues were not subjected to stronger selection than adjacent tissues. Moreover, the CV of the ratio of major to minor shifted on the *x*-axis (Figure 3B), indicating that the allele expression was more variable in tumor tissues, and the significant result of the *t*-test further indicated this (*p*-value = 1.21*E*^−93^). We speculated that the elevated incidence of AEI in tumors resulted from a stochastic process.

### The Incidence of AEI in Genes With Non-synonymous SNPs Is Higher Than That in Genes With Synonymous SNPs in Normal Tissues but Not in Tumor Tissues

To further examine the choice of allele expression in tumors, we grouped genes according to whether the SNPs they carried changed the amino acid sequence, and we analyzed the incidence of AEI in the tumor tissues and adjacent tissues of patients.

In evolutionary analysis, the number of non-synonymous substitutions per non-synonymous site and the number of synonymous substitutions per synonymous site are used to measure selection pressure (Nei and Gojobori, 1986). We analyzed genes with non-synonymous SNPs and synonymous SNPs separately. Our results showed that the proportion of AEI in genes with non-synonymous SNPs was significantly higher than that in genes with synonymous SNPs in adjacent samples (*p-*value = 0.007869 in South Korean patients and *p-*value = 4.26*E*^−9^ in TCGA data); however, this difference could not be observed in tumor samples (*p-*value = 0.348 in TCGA data and *p*-value = 0.5897 in South Korean patients) (Figure 3C and Supplementary Figure S7). The proportions of AEI are shown in Supplementary Tables S1, S2.

In the previous analysis, we found that allele bias has a limited effect on the overall incidence of AEI, but during the evolution of the tumor, natural selection enriches the cell population with growth dominance. Therefore, it is also possible to enrich certain alleles at the chromosome level. Thus, it is still unknown whether the effect of natural selection on the cell population will affect the expression of transcriptome alleles by selecting the proportion of non-synonymous SNP alleles at the chromosome level.

However, we did not observe a significant difference in allele ratio bias in genes with synonymous and non-synonymous SNPs (*p-*value = 0.699 for tumor samples and *p*-value = 0.150 for adjacent samples) (Supplementary Figures S8A,B). Moreover, a difference in the proportion of AEI in adjacent samples was observed after we removed genes that displayed allele ratio bias (Supplementary Figure S8D, *p*-value = 0.01105), but this was not observed in tumor samples (Supplementary Figure S8C, *p*-value = 0.81). We further calculated the proportion of AEI in genes with synonymous and non-synonymous SNPs in data from the “1000 Genomes” project as normal control data since the adjacent tissue may carry tumor-related characteristics. In the “1000 Genomes” project data, we also observed a significant increase in the proportion of AEI in genes with non-synonymous SNPs (*p-*value = 2.38*E*^−29^, Figure 3C and Supplementary Table S5). A previous report showed that non-synonymous RNA editing was rarer than synonymous editing (Xu and Zhang, 2014). Filtering by the dbSNP database would reduce the false rate caused by RNA editing. Furthermore, to demonstrate that the selection characteristics of AEI are not derived from RNA editing, we also removed all the SNPs whose RefSNP alleles were A/G or G/A on sense strands because 94.4% RNA editing was A-to-G (Tang et al., 2012; Shu et al., 2014), and an increase in AEI in genes with non-synonymous SNPs was observed in adjacent samples (Supplementary Figure S9).

Consistent with a higher replacement rate of non-synonymous mutations, the higher incidence of AEI in genes with non-synonymous SNPs in normal tissues indicates that these variations undergo selection pressure. However, in the case of a higher incidence of AEI in tumors, the promotion of AEI in genes with non-synonymous SNPs could not be observed, indicating that instead of being subjected to selection pressure, AEI in tumors reflects increased noise.

## Discussion

The current evolutionary research is based on the theory that natural selection directionally influences population allele frequency. AEI is an ideal tool for the observation of selection characteristics at the transcriptome level. Therefore, in this study, we used AEI to analyze the selection characteristics of lung cancer tissues and normal tissues from different aspects of the transcriptome.

The results from the gene function cluster analysis displayed the differences in the functional adaptation of AEI in tumor tissues and adjacent tissues, with immune-related genes being enriched in the adjacent tissues and DNA-repair-related and stress response genes being enriched in the tumor tissues. Inflammation and lymphocyte infiltration are widely present in the tumor and adjacent tissues (de Martel and Franceschi, 2009; Li et al., 2011). We found immune-related gene expression in both the tumor tissue and the adjacent tissues. However, the biased immune-related gene allele expression found in adjacent tissues was not detected in tumor tissues, which might be the result of cancer immune-editing processes during tumor immune escape (Burnet, 1957; Dunn et al., 2002). Although the enriched AEI in DNA-repair-related and stress response genes could be attributed to the selection pressure in the tumor, the significance of enrichment is much less and the number of genes involved is much fewer than in the adjacent normal tissue.

Conserved genes are generally required for important biological functions, and cancer driver genes often show a higher degree of conservation (Raphael et al., 2014). The negative correlation of conservation and the incidence of AEI in normal tissue conforms to the evolutionary process that the steady-state allele frequency of conserved genes does not change significantly. In contrast, the positive correlation of conservation and the incidence of AEI in tumor tissues implies a stronger selection pressure. However, the association is significant at the nucleotide level but not at the gene level.

SNPs are generally considered to be neutral and relatively stable polymorphisms in the current population. Their influence on regulation is generally also thought to be through transcription factor binding in the transcriptional regulatory region outside the coding region, while stable variation in the exon region directly affects gene regulation. However, in our research, we found that whether the exon SNP changes the amino acid affects the expression ratio of alleles. In the transcriptome data of adjacent tissues and in the 1000 Genomes project data, genes carrying non-synonymous SNPs are significantly more likely to have a higher proportion of AEI than those carrying only synonymous SNPs. In evolutionary analysis, the synonymous substitution rate and the non-synonymous substitution rate of genes are often used as measures of the selection of genes (Nei and Gojobori, 1986). Applying these measures to the transcriptome, our results indicated that this natural selection effect can also be reflected by AEI. In our study of the relationship between AEI and the functional consequence of SNPs, whether the two alleles undergo amino acid changes was the only variable. There is a significant difference in the incidence of AEI, reflecting that exon SNPs can also directly show *cis*-regulatory effects through natural selection.

However, in our analyses, we found that, compared with normal tissues, the correlation between AEI and gene function as well as conservation in tumor tissues showed a weakening trend. In our results, functional fitness can cluster into only very few items. In the correlation analysis between site conservation and the frequency of AEI, association and confidence also showed a decreasing trend in tumor tissues. No significant association of gene conservation and the frequency of AEI was observed in tumors. These results indicate that the selective effect of tumor tissue on the level of AEI is weakened.

Gene expression regulation and stochastic gene expression are the main factors previously thought to have an impact on AEI (Gaur et al., 2013; Eckersley-Maslin et al., 2014). The average value of the ratio of the major allele to the minor allele did not show significant differences between the tumor tissues and the adjacent tissues, but the CV was significantly increased in the tumor tissues. This result suggested that random processes played a more important role in AEI in tumors than gene expression regulation. In summary, our work suggests that the effect of natural selection on SNPs is reflected by AEI and gene expression regulations both in normal tissues and in tumor tissues, whereas in tumor tissues, strong noise in AEI weakens the effect of natural selection.

## Materials and Methods

### Sequencing Data

TCGA sample data: The data used in this study were obtained from the Genomic Data Commons Data Portal^1^ and were identified by screening according to the following criteria: cases in TCGA-LUAD project that have paired primary solid tumor and Solid Tissue Normal for which each sample has both an RNA-seq BAM file and a WXS (or WGS) BAM file. The details for the cases and barcodes are listed in Supplementary Table S1.

### South Korean Patient Data

The samples from different primary lung adenocarcinoma stages from South Korean patients published by Seo et al. (2012) were downloaded from the NCBI Gene Expression Omnibus (GEO)^2^ under accession number GSE40419. We screened 71 patients with cancer with adjacent tissue paired sequencing data and exome sequencing data. Among these patients, 32 (45.07%) were females, and 31 (43.66%) were non-smokers. The publication indexes of the patients are listed in Supplementary Table S2.

1000 Genomes data: Lappalainen et al. (2013) sequenced and analyzed mRNA and miRNAs from lymphocyte cell lines from the 1000 Genomes projects (Geuvadis RNA sequencing project)^3^. We downloaded all the sample data from three populations from Finnish in Finland (FIN), British in England and Scotland (GBR) and Toscani in Italia (TSI) in the Geuvadis RNA sequencing project.

### AEI Analysis

We mapped the RNA-seq data from South Korean patients with tophat2^4^ (Kim et al., 2013) using default parameters, called the SNPs with Samtools^5^ (Li et al., 2009), and then annotated the SNPs with annovar^6^ with the hg19 database (Wang et al., 2010).

In the next-generation sequencing process, the distribution of reads assigned to the reference bases and alternate bases of the hybrid SNP should obey the binomial distribution X ∼ B (n, q), where n is the total number of reads that covered the SNP and q equals 0.5 if there is no AEI. We screened the heterozygous SNPs from the annotated results and denoted Rx as the reference allelic read count and Ax as the alternate allelic read count for the SNPs. We excluded loci with Rx < 4 or Ax < 4 or (Rx + Ax) < 20. If gene X was not displayed under balanced allelic expression, Rx and Ax followed the binomial distribution B [(Rx + Ax), 0.5]. A binomial test was used to screen the AEI genes from the read count. If the *p*-value was less than 0.01, we identified that this gene had AEI.

As the number of effective loci whose DNA and RNA sequencing data both meet the analysis requirements is too small to support subsequent AEI frequency analysis, and from the previous results of the functional consequence of SNPs, the removal of genes that displayed allele ratio bias does not affect; therefore, we used RNA data directly in most analyses.

### Gene Function Cluster Analysis

Gene function cluster analyses for specific AEI genes were carried out with DAVID^7^ (Dennis et al., 2003). We submitted all the non-specific expression genes as background and analyzed the gene sets using goterm_BP_FAT. Entries with a *p-*value < 0.05 were identified as significant.

### Conservative Analysis

The PhastCons conservation data were downloaded from UCSC^8^ and screened for sites in the AEI genes (Karolchik et al., 2014). We analyzed the conservation data and frequency of AEI in these sites with linear regression to examine the associations at the nucleotide level.

We obtained the dN and dS data via orthologous sequencing of *Homo sapiens* genes (GRCh38.p3) to *Macaque Orthologs* in BioMart from Ensembl. AEI genes that had dN/dS data were screened, and the frequency of AEI was examined based on the associations with dN/dS.

## Data Availability Statement

All datasets generated for this study are included in the article/Supplementary Material.

## Author Contributions

JH, XK, and YZ designed the study. JH performed the main bioinformatics analyses. YCZ, MW, YZ, ZX, and MJ provided support. JH, QM, and YHZ wrote the manuscript. All the authors gave final approval for the publication.

## Conflict of Interest

The authors declare that the research was conducted in the absence of any commercial or financial relationships that could be construed as a potential conflict of interest.

## Footnotes

1 https://portal.gdc.cancer.gov/

2 http://www.ncbi.nlm.nih.gov/geo/

3 https://www.internationalgenome.org/category/rnaseq

4 ccb.jhu.edu/software/tophat

5 http://samtools.sourceforge.net/

6 http://annovar.openbioinformatics.org/

7 https://david.ncifcrf.gov/

8 http://hgdownload.cse.ucsc.edu/goldenPath/hg19/phastCons46way/

## Supplementary Material

FIGURE S1 Distribution of the average number of exonic heterozygous SNPs in every gene.

FIGURE S2 Proportions of AEI in adjacent normal tissue (green) and tumor tissue (yellow) samples in South Korean samples. Proportions of AEI in tumor samples were significantly higher than those in adjacent samples (*p*-value = 5.55 × 10^–14^).

FIGURE S3 **(A)** The proportion of allele ratio bias in tumor tissues (yellow) and adjacent tissues (green); the proportion in tumor tissues was significantly larger than that in adjacent tissues (*p-*value = 0.0282). **(B)** The proportion of AEI in genes that did not show allele ratio bias in tumor tissues (yellow) or adjacent tissues (green); the proportion of AEI in tumor tissues still showed a significant increase (*p-*value = 1.06 × 10^–8^).

FIGURE S4 Probability density distributions of the frequency of AEI in genes from tumor tissues and from adjacent tissues.

FIGURE S5 The results showed a negative correlation between site conservation and the frequency of AEI in adjacent tissues (Cor =−0.06, *p*-value = 8.96 × 10^–5^) **(A)** and a positive correlation between site conservation and the frequency of AEI in tumor tissues (Cor = 0.05, *p*-value = 8.06 × 10^–4^) **(B)** in South Korean samples.

FIGURE S6 The result showed a positive correlation between dN/dS and the frequency of AEI in adjacent tissues (Cor = 0.04, *p*-value = 0.01) **(A)**, but a correlation between dN/dS and the frequency of AEI in tumor tissues (*p*-value = 0.91) **(B)** could not be observed.

FIGURE S7 A significant increase in the proportion of AEI in genes with non-synonymous SNPs, compared to those with synonymous SNPs, could be observed in adjacent tissues (*p*-value = 0.00665) but could not be found in tumor tissues (*p*-value = 0.388) in South Korean sample.

FIGURE S8 The proportion of allele ratio bias in tumor tissues and adjacent tissues and the influence of the functional onsequence of SNPs on the allele ratio could not be observed in either tumor tissues (*p-*value = 0.699) **(A)** or adjacent tissues (*p*-value = 0.150) **(B)**. An analysis of the proportions of AEI in genes did not show allele ratio bias with synonymous and non-synonymous SNPs in tumor tissues or adjacent tissues, and the promotion of AEI in genes with non-synonymous SNPs still existed in adjacent tissues (*p-*value = 0.01105) **(D)** but not in tumor tissues (*p*-value = 0.81) **(C)**.

FIGURE S9 After removing potential RNA editing sites and analyzing the proportions of AEI in genes with synonymous and non-synonymous SNPs in tumor tissues and adjacent tissues, the promotion of AEI in genes with non-synonymous SNPs still existed in adjacent tissues (*p-*value = 0.0111) **(B)** but not in tumor tissues (*p-*value = 0.413) **(A)**. The results indicated that RNA editing was not the only factor that changed allele expression.
