# Supplementary figures and images for: Natural Selection on Exonic SNPs Shapes Allelic Expression Imbalance (AEI) Adaptability in Lung Cancer Progression
Source: Front Genet. 2020 Jun 24;11:665. doi: 10.3389/fgene.2020.00665 (PMC7327089; doi:10.3389/fgene.2020.00665)

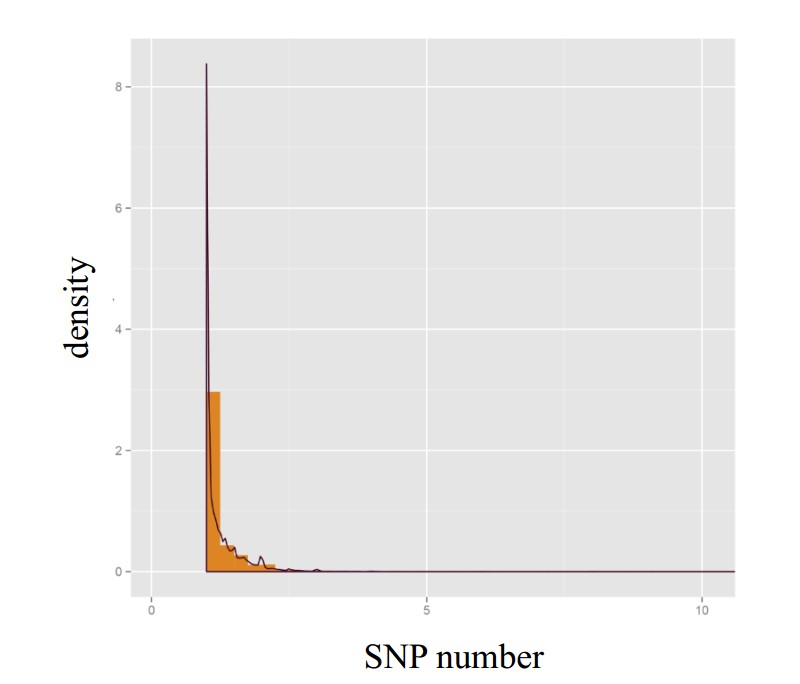

Supplement: FIGURE S1 — Distribution of the average number of exonic heterozygous SNPs in every gene. [file Image_1.JPEG]

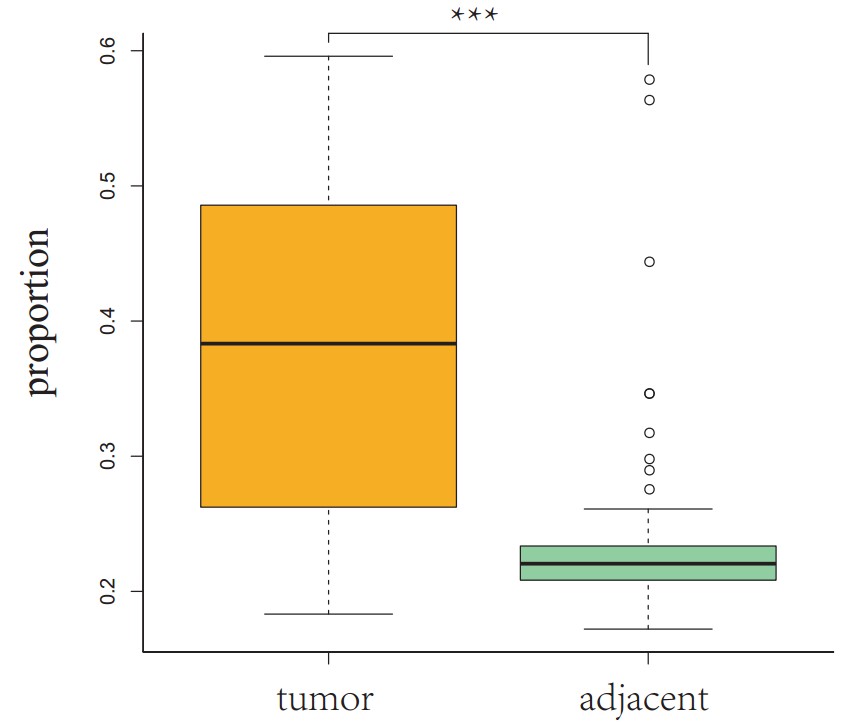

Supplement: FIGURE S2 — Proportions of AEI in adjacent normal tissue (green) and tumor tissue (yellow) samples in South Korean samples. Proportions of AEI in tumor samples were significantly higher than those in adjacent samples (p-value = 5.55 × 10^–14^). [file Image_2.JPEG]

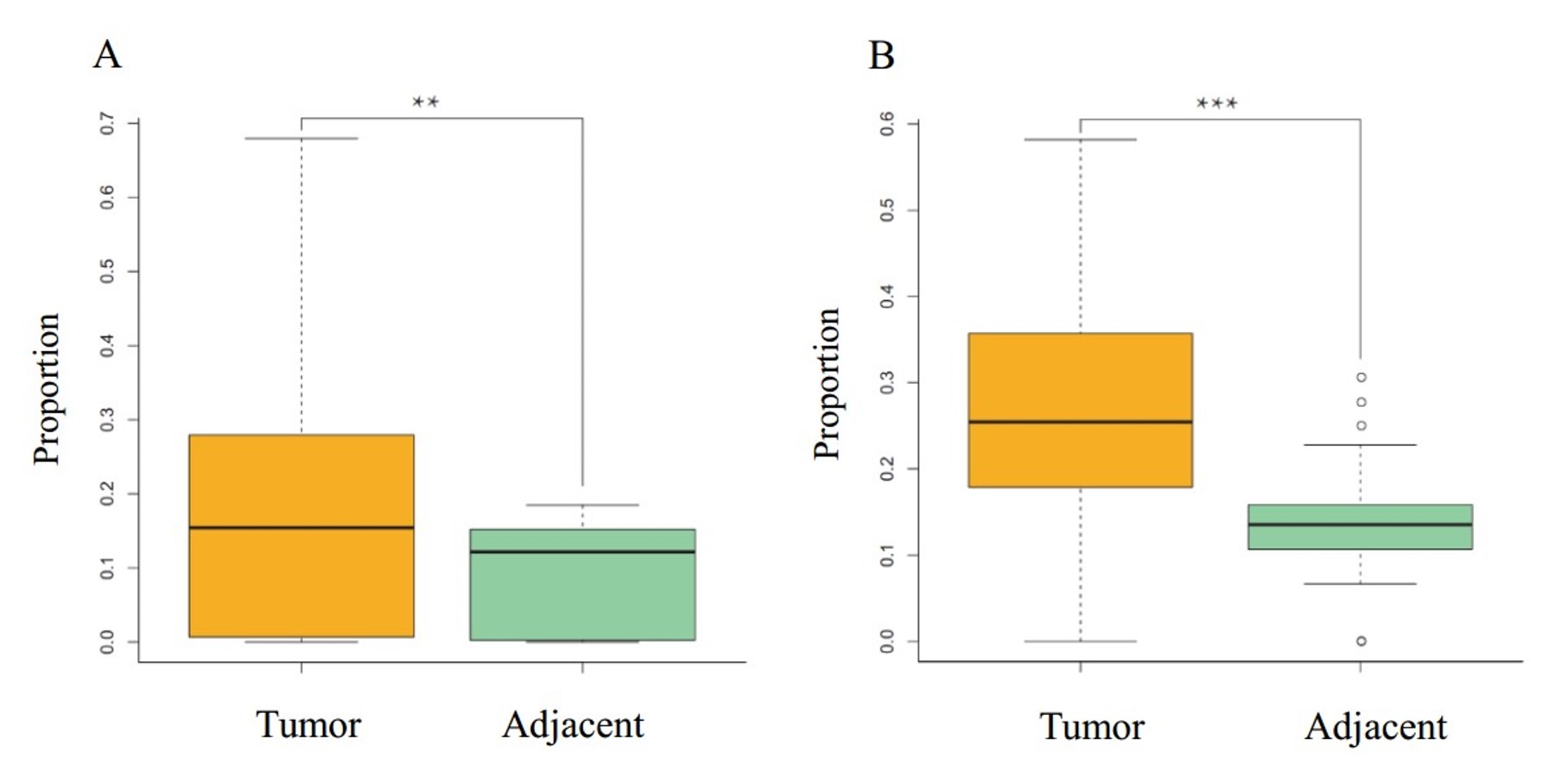

Supplement: FIGURE S3 — (A) The proportion of allele ratio bias in tumor tissues (yellow) and adjacent tissues (green); the proportion in tumor tissues was significantly larger than that in adjacent tissues (p-value = 0.0282). (B) The proportion of AEI in genes that did not show allele ratio bias in tumor tissues (yellow) or adjacent tissues (green); the proportion of AEI in tumor tissues still showed a significant increase (p-value = 1.06 × 10^–8^). [file Image_3.JPEG]

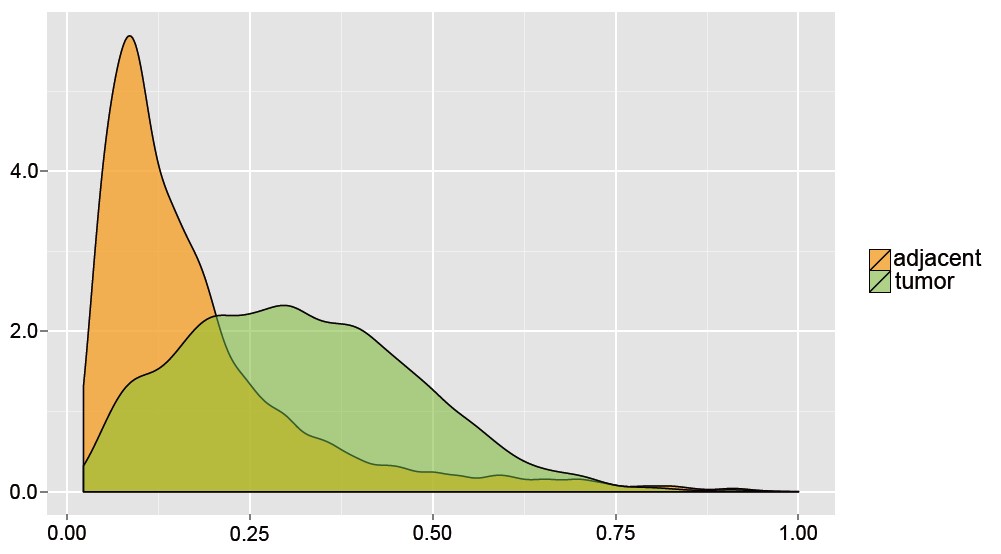

Supplement: FIGURE S4 — Probability density distributions of the frequency of AEI in genes from tumor tissues and from adjacent tissues. [file Image_4.JPEG]

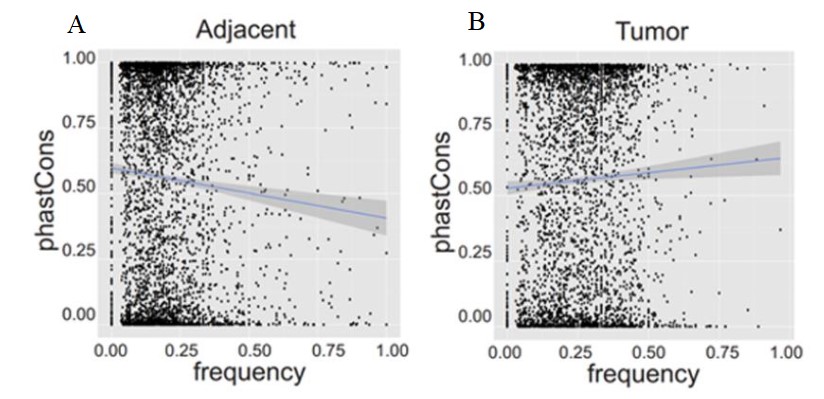

Supplement: FIGURE S5 — The results showed a negative correlation between site conservation and the frequency of AEI in adjacent tissues (Cor =−0.06, p-value = 8.96 × 10^–5^) (A) and a positive correlation between site conservation and the frequency of AEI in tumor tissues (Cor = 0.05, p-value = 8.06 × 10^–4^) (B) in South Korean samples. [file Image_5.JPEG]

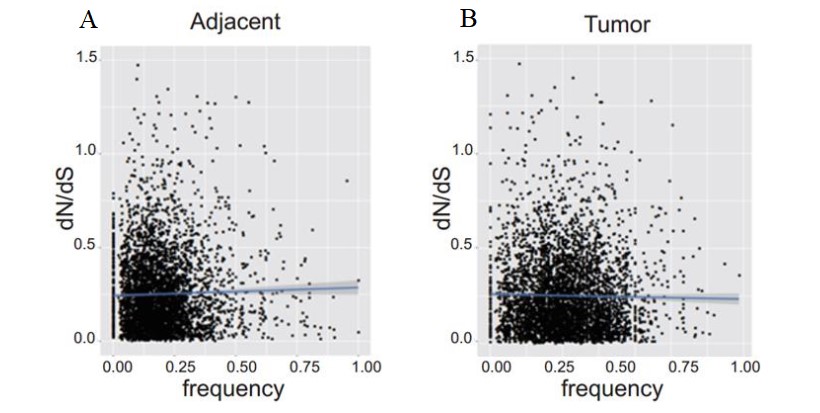

Supplement: FIGURE S6 — The result showed a positive correlation between dN/dS and the frequency of AEI in adjacent tissues (Cor = 0.04, p-value = 0.01) (A), but a correlation between dN/dS and the frequency of AEI in tumor tissues (p-value = 0.91) (B) could not be observed. [file Image_6.JPEG]

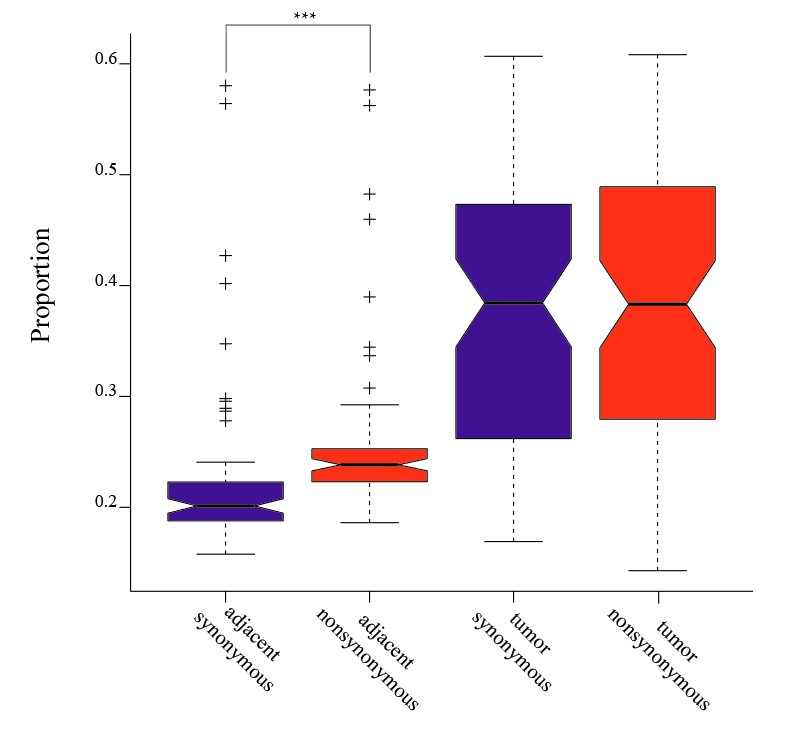

Supplement: FIGURE S7 — A significant increase in the proportion of AEI in genes with non-synonymous SNPs, compared to those with synonymous SNPs, could be observed in adjacent tissues (p-value = 0.00665) but could not be found in tumor tissues (p-value = 0.388) in South Korean sample. [file Image_7.JPEG]

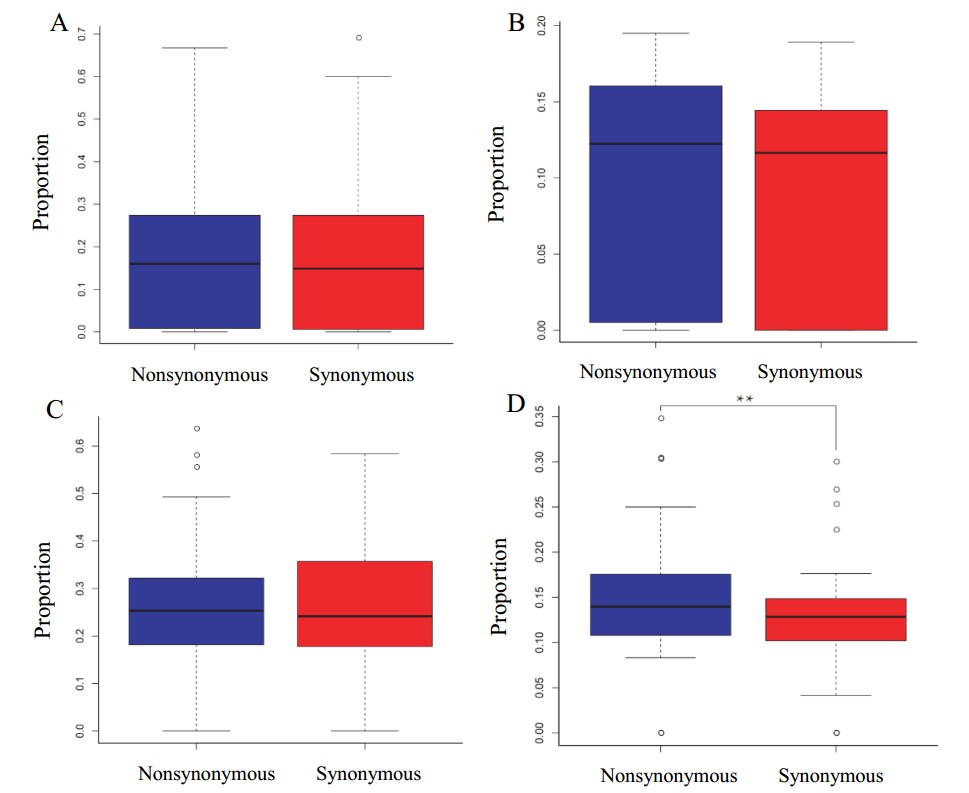

Supplement: FIGURE S8 — The proportion of allele ratio bias in tumor tissues and adjacent tissues and the influence of the functional onsequence of SNPs on the allele ratio could not be observed in either tumor tissues (p-value = 0.699) (A) or adjacent tissues (p-value = 0.150) (B). An analysis of the proportions of AEI in genes did not show allele ratio bias with synonymous and non-synonymous SNPs in tumor tissues or adjacent tissues, and the promotion of AEI in genes with non-synonymous SNPs still existed in adjacent tissues (p-value = 0.01105) (D) but not in tumor tissues (p-value = 0.81) (C). [file Image_8.JPEG]

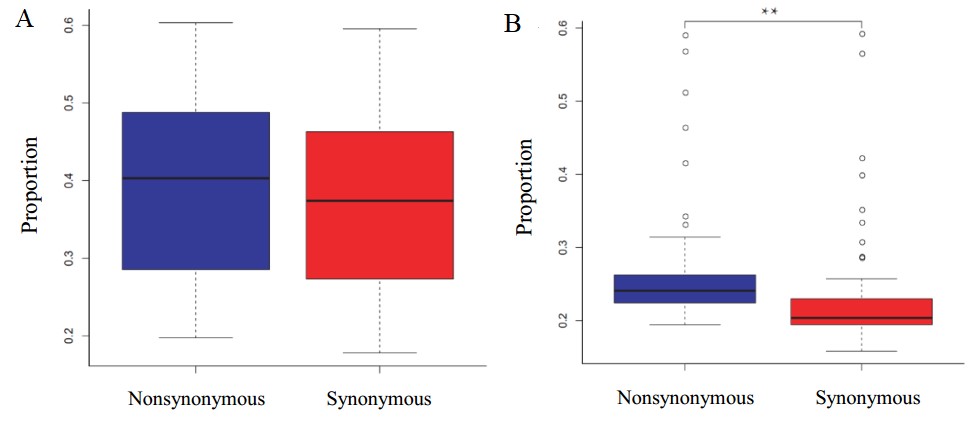

Supplement: FIGURE S9 — After removing potential RNA editing sites and analyzing the proportions of AEI in genes with synonymous and non-synonymous SNPs in tumor tissues and adjacent tissues, the promotion of AEI in genes with non-synonymous SNPs still existed in adjacent tissues (p-value = 0.0111) (B) but not in tumor tissues (p-value = 0.413) (A). The results indicated that RNA editing was not the only factor that changed allele expression. [file Image_9.JPEG]
