# Supplementary material for: Natural Selection on Exonic SNPs Shapes Allelic Expression Imbalance (AEI) Adaptability in Lung Cancer Progression
Source: Front Genet. 2020 Jun 24;11:665. doi: 10.3389/fgene.2020.00665 (PMC7327089; doi:10.3389/fgene.2020.00665)
Supplement: Supplementary file 10 [file Table_1.DOCX]

Supplementary Table 1. Information and AEI proportion of TCGA samples.

| Case ID | Case UUID | Gender | Race | Year of Birth | proportion of AEI | | | | | |
| --- | --- | --- | --- | --- | --- | --- | --- | --- | --- | --- |
|  |  |  |  |  | Tumor | | | Normal | | |
|  |  |  |  |  | Total | Synonymous | Nonsynonymous | Total | Synonymous | Nonsynonymous |
| TCGA-38-4625 | 0ea4920f-f4c8-4590-84b3-eb419824e144 | Female | White | 1936 | 0.53 | 0.525046 | 0.534653 | 0.2 | 0.19213 | 0.204242 |
| TCGA-38-4626 | 2079155a-d91d-4246-a038-01934a580f32 | Female | White | 1946 | 0.22 | 0.200873 | 0.233544 | 0.2 | 0.19241 | 0.205521 |
| TCGA-38-4627 | d717fa33-3c91-4ced-89c6-79ff5542f04c | Female | White | 1939 | 0.27 | 0.268237 | 0.269944 | 0.2 | 0.198451 | 0.204233 |
| TCGA-38-4632 | 875333ab-9048-462d-aaa2-693ad127e3cc | Male | Black Or African American | 1956 | 0.49 | 0.508757 | 0.468416 | 0.16 | 0.153631 | 0.176339 |
| TCGA-44-2655 | 07b5663f-9a54-4462-b6c1-6fc8116b8714 | Female | White | 1944 | 0.3 | 0.290598 | 0.309451 | 0.18 | 0.170381 | 0.197809 |
| TCGA-44-2657 | f40301ba-831e-4afd-9ce8-5f3c1a05ff7e | Female | White | 1935 | 0.4 | 0.398095 | 0.403902 | 0.16 | 0.154585 | 0.169929 |
| TCGA-44-2661 | f3501466-cf32-4866-b5fb-e94dd32341bc | Female | White | 1940 | 0.27 | 0.274149 | 0.273102 | 0.17 | 0.163786 | 0.183099 |
| TCGA-44-2662 | ccda26c1-a6d6-4317-8cf8-8a87e15ce12e | Male | White | 1944 | 0.64 | 0.600605 | 0.657227 | 0.19 | 0.172043 | 0.214765 |
| TCGA-44-2665 | fb79c491-7b01-42ae-8369-8364e442e31b | Female | White | 1954 | 0.38 | 0.266234 | 0.477273 | 0.19 | 0.173882 | 0.206231 |
| TCGA-44-2668 | bab43415-d413-40be-a4c0-2c40a52afe6a | Male | White | 1958 | 0.42 | 0.415574 | 0.427754 | 0.19 | 0.172269 | 0.198227 |
| TCGA-44-3396 | 3bd6badb-27ff-4d8d-b206-4d28dc264862 | Female | White | 1935 | 0.35 | 0.343087 | 0.351542 | 0.2 | 0.198223 | 0.201416 |
| TCGA-44-5645 | ddeacccf-0953-4952-8a4e-c2617f2e7bcc | Female | Black Or African American | 1949 | 0.34 | 0.335535 | 0.344227 | 0.18 | 0.162833 | 0.208361 |
| TCGA-44-6145 | 6144d666-8f77-4efe-95f1-f9e5c4e5e056 | Female | White | 1949 | 0.4 | 0.388566 | 0.409136 | 0.17 | 0.179331 | 0.154011 |
| TCGA-44-6146 | 0c0b610e-fe4c-406d-a5ed-5cc3b11dabf5 | Male | White | 1946 | 0.24 | 0.238422 | 0.234517 | 0.13 | 0.113 | 0.151423 |
| TCGA-44-6147 | 889aec8e-14ba-48d9-8fe1-f2416e82b333 | Female | White | 1943 | 0.75 | 0.636023 | 0.796889 | 0.16 | 0.146577 | 0.180359 |
| TCGA-44-6148 | 8994c8c4-9ea0-46cb-b4a5-0055d7da5bfa | Male | White | 1951 | 0.19 | 0.178275 | 0.200722 | 0.17 | 0.15028 | 0.178498 |
| TCGA-44-6776 | c2a1de2e-6451-4c95-8ce6-263f2b7e6eff | Female | White | 1945 | 0.25 | 0.243304 | 0.250552 | 0.16 | 0.149436 | 0.171737 |
| TCGA-44-6777 | 349e6f38-2c67-4a69-a777-b9173a2a27cf | Female | White | 1921 | 0.31 | 0.323579 | 0.304276 | 0.16 | 0.142237 | 0.173378 |
| TCGA-44-6778 | 5d5cc436-6e57-4d23-a164-7f5153c2e666 | Male | Black Or African American | 1948 | 0.28 | 0.275735 | 0.280981 | 0.15 | 0.136598 | 0.165886 |
| TCGA-49-4490 | 6c3655ca-8ae7-4ad7-b3e4-0c3ec293fba7 | Female | White | 1948 | 0.51 | 0.517647 | 0.5026 | 0.24 | 0.240721 | 0.238042 |
| TCGA-49-4512 | a1e65587-24c1-4b41-92a7-4e1f15fffd78 | Female | White | 1938 | 0.52 | 0.509972 | 0.531707 | 0.29 | 0.265476 | 0.324363 |
| TCGA-49-6742 | 21fb46f9-4bbb-441c-af19-a687e9138344 | Male | White | 1940 | 0.35 | 0.349225 | 0.350478 | 0.17 | 0.158217 | 0.18883 |
| TCGA-49-6743 | a391d49f-a822-460b-981c-6fbe1868ee38 | Female | Female | 1929 | 0.47 | 0.469901 | 0.470389 | 0.17 | 0.143272 | 0.190733 |
| TCGA-49-6744 | 15340dd3-84ae-49fb-989a-eb212f3e73da | Female | White | 1946 | 0.3 | 0.284061 | 0.312647 | 0.16 | 0.146898 | 0.170536 |
| TCGA-49-6745 | 9c7875ad-ab71-4d48-b2e3-e4c7a46393e9 | Male | White | 1929 | 0.38 | 0.378422 | 0.391591 | 0.16 | 0.144641 | 0.184486 |
| TCGA-49-6761 | 4cd3d483-2283-4c6a-a57a-444216119d34 | Female | White | 1941 | 0.54 | 0.560976 | 0.523617 | 0.16 | 0.157568 | 0.171717 |
| TCGA-50-5930 | 368e23f0-e573-4547-bf5a-14080baf737b | Male | White | 1954 | 0.37 | 0.342419 | 0.392123 | 0.17 | 0.154506 | 0.185006 |
| TCGA-50-5931 | 12ccd581-a921-41bc-bcee-4e9be54532cc | Female | White | 1926 | 0.38 | 0.393461 | 0.374046 | 0.17 | 0.151515 | 0.185698 |
| TCGA-50-5932 | ebcba7f2-ce13-4bae-97cd-91a6b1dcd465 | Male | White | 1926 | 0.34 | 0.331658 | 0.347741 | 0.15 | 0.139774 | 0.16129 |
| TCGA-50-5933 | 7a0ea814-f0de-4bc4-a81a-daa000559369 | Male | White | 1929 | 0.45 | 0.434434 | 0.463755 | 0.17 | 0.154418 | 0.189369 |
| TCGA-50-5935 | 100430c8-1446-45c8-af36-b6dbb3ddd0c1 | Female | White | 1920 | 0.48 | 0.48434 | 0.47541 | 0.14 | 0.110136 | 0.162076 |
| TCGA-50-5936 | ef42ae4c-a108-468e-beed-437cf3cf2962 | Male | White | 1949 | 0.39 | 0.398278 | 0.378227 | 0.15 | 0.131374 | 0.1671 |
| TCGA-50-5939 | 12b79b4f-c28c-418e-a1e7-e622f33fdcc2 | Male | White | 1923 | 0.33 | 0.309501 | 0.349336 | 0.16 | 0.128769 | 0.187389 |
| TCGA-50-6595 | 82476d2d-e403-4f6b-8dd6-cc84e3329478 | Female | White | 1935 | 0.53 | 0.54606 | 0.522472 | 0.18 | 0.161337 | 0.202485 |
| TCGA-55-6968 | 83a6ed20-b3cf-48b9-8ed3-a080cd4951fa | Male | White | 1943 | 0.37 | 0.34296 | 0.387939 | 0.14 | 0.132035 | 0.157542 |
| TCGA-55-6969 | d6e274a0-6425-437b-bc07-1225be7808d5 | Male | White | 1952 | 0.49 | 0.499595 | 0.477172 | 0.15 | 0.138298 | 0.161143 |
| TCGA-55-6970 | 7cce924f-12a1-4895-9866-5d4b1869dde6 | Female | White | 1937 | 0.66 | 0.673469 | 0.64392 | 0.16 | 0.151934 | 0.161392 |
| TCGA-55-6971 | 8d0736fe-261c-445c-bfd2-a3ea3ceaf367 | Female | White | 1951 | 0.31 | 0.318681 | 0.304251 | 0.16 | 0.145852 | 0.182469 |
| TCGA-55-6972 | c4d1e105-28b7-48df-abef-fbe09782fdb2 | Male | White | 1933 | 0.4 | 0.407895 | 0.384321 | 0.15 | 0.13039 | 0.162791 |
| TCGA-55-6975 | a046888c-f153-4f35-a1b6-23dd1691a91a | Male | White | 1943 | 0.31 | 0.309305 | 0.318008 | 0.14 | 0.120042 | 0.166856 |
| TCGA-55-6978 | 5134c56f-8286-4ec8-8348-237cee7dad5e | Male | White | 1929 | 0.42 | 0.433708 | 0.406539 | 0.15 | 0.145682 | 0.161734 |
| TCGA-55-6979 | 5af499be-d2b9-4eaf-9a9f-435dccb51917 | Female | White | 1947 | 0.38 | 0.365809 | 0.386018 | 0.16 | 0.142506 | 0.182371 |
| TCGA-55-6980 | 2f0710f4-827b-45b2-9b7e-c27385b481a7 | Male | White | 1950 | 0.34 | 0.318449 | 0.370757 | 0.13 | 0.118463 | 0.142857 |
| TCGA-55-6981 | 0a45f302-5748-48f3-9dc9-66c01843a68e | Female | White | 1952 | 0.44 | 0.435233 | 0.449871 | 0.14 | 0.125551 | 0.148681 |
| TCGA-55-6982 | 35cb7841-9b09-465a-90c5-e3b8a9faad49 | Female | White | 1925 | 0.48 | 0.496114 | 0.470207 | 0.16 | 0.142361 | 0.174954 |
| TCGA-55-6983 | 54a0e4d7-c274-4569-9c25-45bbe26d7f21 | Male | White | 1924 | 0.27 | 0.255178 | 0.283804 | 0.15 | 0.122693 | 0.171202 |
| TCGA-55-6984 | f063ddbb-1668-40df-9ec2-b0a23ca2c389 | Female | White | 1935 | 0.23 | 0.212878 | 0.250329 | 0.18 | 0.16189 | 0.188571 |
| TCGA-55-6985 | e5cb0c86-8fe2-4cfc-b32b-e8ec3839ffc4 | Female | White | 1946 | 0.34 | 0.321083 | 0.367428 | 0.21 | 0.191443 | 0.220434 |
| TCGA-55-6986 | 028e99e9-5b9a-4954-bb6e-6d4709a3cea8 | Female | White | 1930 | 0.27 | 0.262774 | 0.271028 | 0.18 | 0.16712 | 0.201393 |
| TCGA-73-4676 | 195a5afb-b79f-44d2-9d12-884487630c2b | Male | White | 1965 | 0.61 | 0.62 | 0.592593 | 0.19 | 0.15777 | 0.219945 |
| TCGA-91-6828 | 9536e32d-2707-48d2-a36d-08c521665bb9 | Male | White | 1941 | 0.31 | 0.324352 | 0.298097 | 0.14 | 0.135472 | 0.15331 |
| TCGA-91-6829 | 1427cd18-5ad3-491a-9981-908e31ae49db | Male | White | 1923 | 0.58 | 0.596279 | 0.558161 | 0.16 | 0.134328 | 0.19227 |
| TCGA-91-6831 | db0b9e63-4272-4f29-bf0e-1ec0fe79a9d7 | Male | White | 1935 | 0.46 | 0.448313 | 0.469565 | 0.13 | 0.101877 | 0.159218 |
| TCGA-91-6835 | 2f09479f-87fc-4c34-8e2c-333e970a3681 | Female | White | 1929 | 0.46 | 0.482938 | 0.431587 | 0.15 | 0.137133 | 0.152921 |
| TCGA-91-6836 | 0b31dbdf-0623-48e4-a0ec-017650dceda7 | Female | White | 1958 | 0.59 | 0.58567 | 0.591039 | 0.16 | 0.149425 | 0.169492 |
| TCGA-91-6847 | 97831d28-ab41-4c18-bfc2-c4c6bc757d13 | Female | White | 1940 | 0.65 | 0.657283 | 0.647386 | 0.14 | 0.138393 | 0.14411 |
| TCGA-91-6849 | 953a908d-0993-42f6-853b-512611d19a2c | Female | Black Or African American | 1934 | 0.25 | 0.24055 | 0.257844 | 0.14 | 0.124143 | 0.155462 |
