# Supplementary material for: Natural Selection on Exonic SNPs Shapes Allelic Expression Imbalance (AEI) Adaptability in Lung Cancer Progression
Source: Front Genet. 2020 Jun 24;11:665. doi: 10.3389/fgene.2020.00665 (PMC7327089; doi:10.3389/fgene.2020.00665)
Supplement: Supplementary file 11 [file Table_2.DOCX]

Supplementary Table 2．AEI proportion of South Korean samples

| Publication  _index | Age | Gender (1=male,  2=female) | Smoking_status (0=neverSmoker;  1=smoker;  2=current_smoker;  3=unknown) | Stage | Proportion of AEI | | | | | |
| --- | --- | --- | --- | --- | --- | --- | --- | --- | --- | --- |
|  |  |  |  |  | Tumor | | | Adnacent | | |
|  |  |  |  |  | Total | Synonymous | Nonsynonymous | Total | Synonymous | Nonsynonymous |
| LC_C1 | 54 | 1 | 1 | 1A | 0.396267 | 0.390244 | 0.402361 | 0.239377 | 0.229765 | 0.234375 |
| LC_C10 | 54 | 1 | 1 | 1A | 0.58764 | 0.567285 | 0.606754 | 0.248352 | 0.204082 | 0.225397 |
| LC_C11 | 54 | 1 | 1 | 1A | 0.436986 | 0.45 | 0.425 | 0.238426 | 0.199199 | 0.217391 |
| LC_C12 | 54 | 1 | 1 | 1A | 0.332689 | 0.383202 | 0.283904 | 0.241463 | 0.190987 | 0.214612 |
| LC_C14 | 54 | 1 | 1 | 1A | 0.308693 | 0.31728 | 0.300662 | 0.264151 | 0.181467 | 0.222222 |
| LC_C16 | 54 | 2 | 0 | 1A | 0.256297 | 0.275441 | 0.239718 | 0.237028 | 0.182112 | 0.208333 |
| LC_C17 | 64 | 2 | 0 | 1A | 0.231815 | 0.246173 | 0.219405 | 0.244576 | 0.204282 | 0.223419 |
| LC_C18 | 68 | 2 | 1 | 1B | 0.43559 | 0.408284 | 0.463592 | 0.252652 | 0.228672 | 0.24011 |
| LC_C19 | 40 | 2 | 0 | 4 | 0.387662 | 0.37741 | 0.396806 | 0.246249 | 0.217749 | 0.231489 |
| LC_C2 | 40 | 2 | 0 | 4 | 0.227025 | 0.231383 | 0.22243 | 0.229167 | 0.213831 | 0.221679 |
| LC_C20 | 65 | 1 | 1 | 1A | 0.496324 | 0.495146 | 0.497305 | 0.222112 | 0.214418 | 0.218121 |
| LC_C21 | 42 | 1 | 1 | 1B | 0.480595 | 0.461538 | 0.5 | 0.222609 | 0.202267 | 0.212451 |
| LC_C22 | 73 | 1 | 1 | 1A | 0.434643 | 0.422753 | 0.444709 | 0.272572 | 0.278049 | 0.275374 |
| LC_C24 | 66 | 2 | 0 | 1A | 0.275424 | 0.286969 | 0.264666 | 0.215967 | 0.195812 | 0.206004 |
| LC_C25 | 66 | 1 | 1 | 1A | 0.338795 | 0.338164 | 0.339387 | 0.243115 | 0.217195 | 0.229842 |
| LC_C28 | 59 | 2 | 0 | NA | 0.428753 | 0.473118 | 0.388889 | 0.241538 | 0.226858 | 0.233592 |
| LC_C29 | 60 | 2 | 0 | 4 | 0.265688 | 0.273504 | 0.258794 | 0.224401 | 0.218901 | 0.221483 |
| LC_C3 | 60 | 2 | 0 | 4 | 0.284339 | 0.313078 | 0.257179 | 0.263645 | 0.191214 | 0.226137 |
| LC_C30 | 38 | 2 | 0 | 1A | 0.368821 | 0.357333 | 0.379227 | 0.242781 | 0.226708 | 0.234613 |
| LC_C31 | 61 | 2 | 0 | 1A | 0.220244 | 0.22334 | 0.217544 | 0.18619 | 0.159468 | 0.172112 |
| LC_C33 | 62 | 1 | 3 | NA | 0.490252 | 0.482326 | 0.49827 | 0.292494 | 0.286713 | 0.28946 |
| LC_C34 | 73 | 1 | 1 | 1B | 0.417808 | 0.416667 | 0.418969 | 0.22964 | 0.213731 | 0.221565 |
| LC_C35 | 60 | 1 | 2 | 2A | 0.260908 | 0.261026 | 0.260796 | 0.222222 | 0.181818 | 0.200978 |
| LC_C36 | 56 | 2 | 0 | 1A | 0.534632 | 0.52027 | 0.547917 | 0.344492 | 0.347505 | 0.346116 |
| LC_C5 | 56 | 2 | 0 | 1A | 0.25924 | 0.268374 | 0.251004 | 0.24 | 0.20508 | 0.22156 |
| LC_C7 | 56 | 2 | 0 | 1A | 0.487265 | 0.501126 | 0.473856 | 0.258021 | 0.240091 | 0.248314 |
| LC_C9 | 56 | 2 | 0 | 1A | 0.530375 | 0.522374 | 0.539884 | 0.252874 | 0.212092 | 0.232502 |
| LC_S10 | 56 | 2 | 0 | 1A | 0.368184 | 0.415507 | 0.320242 | 0.22604 | 0.167082 | 0.195323 |
| LC_S11 | 56 | 2 | 0 | 1A | 0.584295 | 0.574028 | 0.596172 | 0.217638 | 0.183502 | 0.200908 |
| LC_S13 | 56 | 2 | 0 | 1A | 0.322835 | 0.311475 | 0.333333 | 0.271886 | 0.236269 | 0.25439 |
| LC_S14 | 56 | 2 | 0 | 1A | 0.20332 | 0.222222 | 0.185484 | 0.576446 | 0.580262 | 0.578447 |
| LC_S15 | 56 | 2 | 0 | 1A | 0.489455 | 0.495172 | 0.483077 | 0.217273 | 0.187557 | 0.202462 |
| LC_S16 | 56 | 2 | 0 | 1A | 0.261693 | 0.29717 | 0.229958 | 0.281356 | 0.240772 | 0.260961 |
| LC_S17 | 73 | 1 | 1 | 1A | 0.386 | 0.388773 | 0.38343 | 0.248299 | 0.184391 | 0.216482 |
| LC_S18 | 76 | 1 | 1 | 3A | 0.485723 | 0.497291 | 0.472527 | 0.218371 | 0.199336 | 0.208651 |
| LC_S19 | 62 | 1 | 1 | 1A | 0.595917 | 0.608239 | 0.58221 | 0.228522 | 0.193047 | 0.211342 |
| LC_S20 | 58 | 1 | 1 | 1B | 0.379924 | 0.382911 | 0.376796 | 0.215014 | 0.190391 | 0.202451 |
| LC_S21 | 52 | 1 | 2 | 1A | 0.314597 | 0.333607 | 0.294773 | 0.227199 | 0.178482 | 0.203131 |
| LC_S22 | 67 | 2 | 0 | 1B | 0.224737 | 0.244015 | 0.205047 | 0.238131 | 0.199513 | 0.219531 |
| LC_S23 | 62 | 2 | 0 | 1B | 0.389395 | 0.384458 | 0.394826 | 0.45974 | 0.427034 | 0.443674 |
| LC_S24 | 70 | 2 | 0 | 2A | 0.450363 | 0.463749 | 0.435294 | 0.223868 | 0.195353 | 0.209524 |
| LC_S25 | 78 | 1 | 1 | 3A | 0.57918 | 0.572358 | 0.587271 | 0.223147 | 0.193798 | 0.208721 |
| LC_S26 | 70 | 2 | 0 | 1B | 0.458987 | 0.472401 | 0.445104 | 0.234043 | 0.235294 | 0.234679 |
| LC_S27 | 65 | 1 | 1 | 1A | 0.352081 | 0.370273 | 0.333631 | 0.307536 | 0.289264 | 0.297771 |
| LC_S28 | 63 | 1 | 0 | 1B | 0.250356 | 0.27062 | 0.228188 | 0.231939 | 0.201258 | 0.216853 |
| LC_S29 | 59 | 1 | 2 | 3A | 0.528994 | 0.528692 | 0.529316 | 0.218227 | 0.157715 | 0.188265 |
| LC_S30 | 66 | 2 | 0 | 1B | 0.242934 | 0.25723 | 0.228528 | 0.230708 | 0.183456 | 0.207425 |
| LC_S31 | 56 | 1 | 1 | 2B | 0.53809 | 0.531989 | 0.545092 | 0.226234 | 0.190476 | 0.209018 |
| LC_S32 | 64 | 1 | 1 | 1A | 0.581738 | 0.57388 | 0.590081 | 0.220695 | 0.186275 | 0.203576 |
| LC_S33 | 66 | 2 | 0 | 1A | 0.18807 | 0.204643 | 0.169113 | 0.246025 | 0.203282 | 0.225567 |
| LC_S34 | 45 | 2 | 0 | 1B | 0.523419 | 0.526198 | 0.520392 | 0.243725 | 0.19883 | 0.221628 |
| LC_S35 | 82 | 1 | 1 | 4 | 0.438962 | 0.447303 | 0.430192 | 0.389731 | 0.298131 | 0.346324 |
| LC_S36 | 74 | 2 | 0 | 1B | 0.243922 | 0.261453 | 0.226066 | 0.212428 | 0.202541 | 0.207568 |
| LC_S37 | 69 | 1 | 1 | 2B | 0.183215 | 0.189331 | 0.176471 | 0.262246 | 0.218002 | 0.241135 |
| LC_S39 | 58 | 1 | 1 | 2B | 0.449834 | 0.448914 | 0.450813 | 0.251935 | 0.20542 | 0.228869 |
| LC_S40 | 47 | 1 | 2 | 2B | 0.285363 | 0.297872 | 0.273277 | 0.218519 | 0.179856 | 0.198905 |
| LC_S41 | 55 | 2 | 1 | 3A | 0.243863 | 0.283648 | 0.207179 | 0.231653 | 0.197694 | 0.214072 |
| LC_S42 | 62 | 2 | 0 | 3B | 0.380608 | 0.376712 | 0.384396 | 0.247436 | 0.197564 | 0.223173 |
| LC_S43 | 68 | 2 | 0 | 1B | 0.412239 | 0.424038 | 0.400749 | 0.254358 | 0.209184 | 0.232568 |
| LC_S44 | 75 | 2 | 0 | 1A | 0.215134 | 0.237949 | 0.193887 | 0.238385 | 0.179604 | 0.208984 |
| LC_S45 | 72 | 1 | 0 | 1A | 0.330387 | 0.352018 | 0.309368 | 0.223043 | 0.196844 | 0.210056 |
| LC_S46 | 66 | 1 | 0 | 1B | 0.476621 | 0.482963 | 0.470046 | 0.231527 | 0.212295 | 0.221903 |
| LC_S47 | 75 | 2 | 0 | 3B | 0.255837 | 0.247413 | 0.264854 | 0.222785 | 0.180556 | 0.201968 |
| LC_S48 | 48 | 2 | 0 | 1B | 0.279901 | 0.29602 | 0.264059 | 0.233499 | 0.188156 | 0.211447 |
| LC_S49 | 66 | 2 | 0 | 1A | 0.464422 | 0.515152 | 0.407166 | 0.562408 | 0.564173 | 0.56332 |
| LC_S50 | 64 | 2 | 0 | 1B | 0.28744 | 0.294059 | 0.281132 | 0.202381 | 0.181668 | 0.191873 |
| LC_S51 | 64 | 1 | 2 | 2A | 0.508631 | 0.512575 | 0.504447 | 0.216745 | 0.188285 | 0.202992 |
| LC_S52 | 82 | 2 | 1 | 1A | 0.262317 | 0.261299 | 0.263224 | 0.220426 | 0.181818 | 0.201744 |
| LC_S6 | 66 | 2 | 0 | 1B | 0.485884 | 0.537391 | 0.417722 | 0.336831 | 0.295662 | 0.317113 |
| LC_S9 | 69 | 1 | 0 | 1B | 0.553279 | 0.581988 | 0.518209 | 0.251693 | 0.186263 | 0.219484 |
