# Supplementary material for: Natural Selection on Exonic SNPs Shapes Allelic Expression Imbalance (AEI) Adaptability in Lung Cancer Progression
Source: Front Genet. 2020 Jun 24;11:665. doi: 10.3389/fgene.2020.00665 (PMC7327089; doi:10.3389/fgene.2020.00665)
Supplement: Supplementary file 14 [file Table_5.DOCX]

| Sample_id | AEI proportion of synonymous SNPs | AEI proportion of missense SNPs | AEI proportion of total SNPs |
| --- | --- | --- | --- |
| HG00096 | 0.100166 | 0.142744 | 0.121971 |
| HG00097 | 0.152254 | 0.15457 | 0.153467 |
| HG00099 | 0.145516 | 0.129464 | 0.137706 |
| HG00111 | 0.146545 | 0.160273 | 0.153328 |
| HG00112 | 0.108507 | 0.139706 | 0.124579 |
| HG00114 | 0.126325 | 0.123404 | 0.124837 |
| HG00115 | 0.117201 | 0.157179 | 0.137114 |
| HG00116 | 0.15264 | 0.16734 | 0.160065 |
| HG00117 | 0.157524 | 0.170439 | 0.164507 |
| HG00118 | 0.160128 | 0.143297 | 0.151551 |
| HG00119 | 0.13245 | 0.162992 | 0.148103 |
| HG00120 | 0.128814 | 0.139535 | 0.134097 |
| HG00121 | 0.125356 | 0.141689 | 0.133705 |
| HG00122 | 0.142745 | 0.147882 | 0.145289 |
| HG00123 | 0.153344 | 0.16129 | 0.15734 |
| HG00124 | 0.16559 | 0.165473 | 0.165531 |
| HG00125 | 0.1113 | 0.123928 | 0.117886 |
| HG00126 | 0.141239 | 0.166142 | 0.153431 |
| HG00127 | 0.144606 | 0.173333 | 0.15959 |
| HG00128 | 0.122502 | 0.149956 | 0.136205 |
| HG00129 | 0.127583 | 0.138136 | 0.133014 |
| HG00130 | 0.143113 | 0.165074 | 0.154253 |
| HG00131 | 0.142361 | 0.153971 | 0.148365 |
| HG00132 | 0.160745 | 0.168046 | 0.164435 |
| HG00133 | 0.137739 | 0.149445 | 0.143387 |
| HG00134 | 0.161029 | 0.174427 | 0.167917 |
| HG00135 | 0.105858 | 0.118201 | 0.111975 |
| HG00136 | 0.116183 | 0.169374 | 0.143715 |
| HG00137 | 0.122034 | 0.136049 | 0.128963 |
| HG00138 | 0.140934 | 0.171326 | 0.156095 |
| HG00139 | 0.164057 | 0.158931 | 0.161419 |
| HG00141 | 0.170597 | 0.17484 | 0.172826 |
| HG00142 | 0.152062 | 0.173639 | 0.163307 |
| HG00143 | 0.141694 | 0.139338 | 0.140483 |
| HG00145 | 0.135579 | 0.151708 | 0.14378 |
| HG00146 | 0.148387 | 0.145497 | 0.146908 |
| HG00148 | 0.150327 | 0.180113 | 0.165185 |
| HG00149 | 0.120196 | 0.16328 | 0.143022 |
| HG00150 | 0.157759 | 0.157209 | 0.157468 |
| HG00151 | 0.105528 | 0.153191 | 0.128682 |
| HG00152 | 0.171223 | 0.169836 | 0.170497 |
| HG00154 | 0.162321 | 0.17352 | 0.167983 |
| HG00155 | 0.103952 | 0.134794 | 0.119524 |
| HG00156 | 0.147208 | 0.182848 | 0.165426 |
| HG00157 | 0.191083 | 0.185336 | 0.18815 |
| HG00158 | 0.134213 | 0.158364 | 0.146545 |
| HG00159 | 0.152191 | 0.147293 | 0.149741 |
| HG00160 | 0.128205 | 0.168033 | 0.147363 |
| HG00171 | 0.148816 | 0.194993 | 0.170109 |
| HG00173 | 0.132056 | 0.155702 | 0.143382 |
| HG00174 | 0.134969 | 0.168842 | 0.151965 |
| HG00176 | 0.141258 | 0.159262 | 0.150382 |
| HG00177 | 0.16766 | 0.155214 | 0.161277 |
| HG00178 | 0.208 | 0.211524 | 0.20976 |
| HG00179 | 0.140695 | 0.137097 | 0.138901 |
| HG00180 | 0.132806 | 0.167559 | 0.150467 |
| HG00181 | 0.1376 | 0.13186 | 0.13466 |
| HG00182 | 0.123128 | 0.119703 | 0.121319 |
| HG00183 | 0.149676 | 0.175137 | 0.162624 |
| HG00185 | 0.134352 | 0.163189 | 0.149323 |
| HG00186 | 0.11134 | 0.144295 | 0.127146 |
| HG00187 | 0.156773 | 0.156904 | 0.156841 |
| HG00188 | 0.1488 | 0.156589 | 0.152756 |
| HG00189 | 0.125696 | 0.136651 | 0.131193 |
| HG00231 | 0.152364 | 0.149587 | 0.150935 |
| HG00232 | 0.109195 | 0.135317 | 0.122244 |
| HG00233 | 0.144868 | 0.182312 | 0.164197 |
| HG00234 | 0.150422 | 0.172233 | 0.162191 |
| HG00235 | 0.134048 | 0.152212 | 0.143175 |
| HG00236 | 0.18614 | 0.195884 | 0.191148 |
| HG00238 | 0.147128 | 0.151446 | 0.149323 |
| HG00239 | 0.139793 | 0.163349 | 0.152011 |
| HG00240 | 0.163313 | 0.175055 | 0.169358 |
| HG00242 | 0.147788 | 0.158787 | 0.153265 |
| HG00243 | 0.126246 | 0.163265 | 0.145278 |
| HG00244 | 0.142412 | 0.168992 | 0.155728 |
| HG00245 | 0.148754 | 0.157709 | 0.153447 |
| HG00246 | 0.155172 | 0.15316 | 0.15414 |
| HG00247 | 0.157804 | 0.171975 | 0.165153 |
| HG00249 | 0.138943 | 0.153218 | 0.145927 |
| HG00250 | 0.128141 | 0.131518 | 0.129891 |
| HG00251 | 0.13858 | 0.184458 | 0.161538 |
| HG00252 | 0.141487 | 0.151681 | 0.14664 |
| HG00253 | 0.150843 | 0.186005 | 0.16844 |
| HG00255 | 0.142516 | 0.158842 | 0.15075 |
| HG00256 | 0.144426 | 0.167468 | 0.15625 |
| HG00257 | 0.121065 | 0.136508 | 0.128852 |
| HG00258 | 0.137304 | 0.154086 | 0.14595 |
| HG00259 | 0.179059 | 0.19346 | 0.186648 |
| HG00260 | 0.148235 | 0.163882 | 0.156003 |
| HG00261 | 0.159432 | 0.183172 | 0.17198 |
| HG00262 | 0.161633 | 0.183891 | 0.17316 |
| HG00263 | 0.1272 | 0.153214 | 0.140649 |
| HG00264 | 0.141144 | 0.147251 | 0.144182 |
| HG00265 | 0.176895 | 0.176573 | 0.176732 |
| HG00266 | 0.156947 | 0.170415 | 0.163652 |
| HG00267 | 0.132923 | 0.149329 | 0.141323 |
| HG00268 | 0.151973 | 0.16148 | 0.156723 |
| HG00269 | 0.15608 | 0.146694 | 0.151691 |
| HG00271 | 0.153118 | 0.166416 | 0.159923 |
| HG00272 | 0.138889 | 0.145344 | 0.14224 |
| HG00273 | 0.132853 | 0.159551 | 0.145621 |
| HG00274 | 0.137239 | 0.153349 | 0.145272 |
| HG00275 | 0.175494 | 0.182955 | 0.179245 |
| HG00276 | 0.165873 | 0.181887 | 0.174048 |
| HG00277 | 0.137047 | 0.171456 | 0.153563 |
| HG00278 | 0.126747 | 0.159735 | 0.143125 |
| HG00280 | 0.110649 | 0.119142 | 0.114994 |
| HG00281 | 0.159664 | 0.161429 | 0.160537 |
| HG00282 | 0.144454 | 0.169124 | 0.156736 |
| HG00284 | 0.158375 | 0.165791 | 0.162269 |
| HG00285 | 0.149826 | 0.169308 | 0.159778 |
| HG00306 | 0.133663 | 0.155378 | 0.14471 |
| HG00308 | 0.135164 | 0.154015 | 0.144397 |
| HG00309 | 0.1721 | 0.216963 | 0.196127 |
| HG00310 | 0.152051 | 0.153369 | 0.152723 |
| HG00311 | 0.119005 | 0.155498 | 0.137555 |
| HG00312 | 0.164236 | 0.187986 | 0.1766 |
| HG00313 | 0.147476 | 0.172385 | 0.159562 |
| HG00315 | 0.13923 | 0.170616 | 0.155207 |
| HG00319 | 0.137694 | 0.142741 | 0.140285 |
| HG00320 | 0.148741 | 0.158106 | 0.153305 |
| HG00321 | 0.151764 | 0.1783 | 0.16555 |
| HG00323 | 0.126254 | 0.131175 | 0.128692 |
| HG00324 | 0.162299 | 0.163017 | 0.162666 |
| HG00325 | 0.145174 | 0.169881 | 0.158078 |
| HG00326 | 0.140244 | 0.149365 | 0.144851 |
| HG00327 | 0.158915 | 0.169 | 0.163878 |
| HG00328 | 0.193297 | 0.178516 | 0.185875 |
| HG00329 | 0.110687 | 0.10989 | 0.110317 |
| HG00330 | 0.145916 | 0.160793 | 0.153641 |
| HG00331 | 0.133077 | 0.152225 | 0.14258 |
| HG00332 | 0.157165 | 0.174905 | 0.166093 |
| HG00334 | 0.13486 | 0.151754 | 0.143165 |
| HG00335 | 0.144338 | 0.162471 | 0.153606 |
| HG00336 | 0.148413 | 0.173811 | 0.161227 |
| HG00337 | 0.155263 | 0.140367 | 0.147982 |
| HG00338 | 0.185304 | 0.182239 | 0.183746 |
| HG00339 | 0.156557 | 0.141234 | 0.148858 |
| HG00341 | 0.151575 | 0.163673 | 0.157582 |
| HG00342 | 0.14939 | 0.176681 | 0.163469 |
| HG00343 | 0.161983 | 0.167173 | 0.164687 |
| HG00344 | 0.170648 | 0.146721 | 0.158445 |
| HG00345 | 0.129058 | 0.14688 | 0.138145 |
| HG00346 | 0.189655 | 0.188156 | 0.188889 |
| HG00349 | 0.160635 | 0.141991 | 0.151224 |
| HG00350 | 0.155063 | 0.159907 | 0.157372 |
| HG00351 | 0.114226 | 0.143001 | 0.128834 |
| HG00353 | 0.151575 | 0.133937 | 0.142386 |
| HG00355 | 0.169135 | 0.173913 | 0.171571 |
| HG00356 | 0.164706 | 0.186873 | 0.175875 |
| HG00358 | 0.130285 | 0.172757 | 0.151926 |
| HG00359 | 0.155226 | 0.154188 | 0.154694 |
| HG00360 | 0.129148 | 0.144942 | 0.136724 |
| HG00361 | 0.1408 | 0.163326 | 0.152484 |
| HG01334 | 0.168018 | 0.170613 | 0.169352 |
| HG01789 | 0.136071 | 0.141762 | 0.138987 |
| HG01790 | 0.107814 | 0.135347 | 0.120735 |
| HG01791 | 0.179104 | 0.211268 | 0.195652 |
| HG02215 | 0.155395 | 0.16628 | 0.160947 |
| NA20502 | 0.137931 | 0.154918 | 0.146746 |
| NA20503 | 0.140857 | 0.157938 | 0.149425 |
| NA20504 | 0.15847 | 0.186933 | 0.172727 |
| NA20505 | 0.162471 | 0.162031 | 0.162241 |
| NA20506 | 0.126249 | 0.144435 | 0.135645 |
| NA20507 | 0.117428 | 0.143369 | 0.130653 |
| NA20508 | 0.151745 | 0.155198 | 0.153502 |
| NA20509 | 0.190108 | 0.217617 | 0.205074 |
| NA20510 | 0.089537 | 0.146341 | 0.117192 |
| NA20512 | 0.112676 | 0.154839 | 0.133953 |
| NA20513 | 0.150204 | 0.177528 | 0.164453 |
| NA20514 | 0.119931 | 0.136574 | 0.128717 |
| NA20515 | 0.154905 | 0.165407 | 0.160221 |
| NA20516 | 0.147495 | 0.154917 | 0.151325 |
| NA20517 | 0.14726 | 0.157322 | 0.152349 |
| NA20518 | 0.141056 | 0.153392 | 0.147429 |
| NA20519 | 0.136071 | 0.163541 | 0.150291 |
| NA20520 | 0.129925 | 0.132231 | 0.131019 |
| NA20521 | 0.135636 | 0.131601 | 0.133613 |
| NA20524 | 0.177258 | 0.159126 | 0.167877 |
| NA20525 | 0.102282 | 0.134677 | 0.118861 |
| NA20527 | 0.161753 | 0.179608 | 0.170751 |
| NA20528 | 0.166269 | 0.202096 | 0.184728 |
| NA20529 | 0.177798 | 0.154762 | 0.166364 |
| NA20530 | 0.187739 | 0.199362 | 0.193435 |
| NA20531 | 0.151144 | 0.170243 | 0.161327 |
| NA20532 | 0.152409 | 0.192825 | 0.173546 |
| NA20534 | 0.159574 | 0.155421 | 0.157418 |
| NA20535 | 0.145 | 0.158809 | 0.15193 |
| NA20536 | 0.12775 | 0.144737 | 0.136364 |
| NA20537 | 0.141627 | 0.144153 | 0.142857 |
| NA20538 | 0.122167 | 0.154085 | 0.13774 |
| NA20539 | 0.134011 | 0.154189 | 0.144283 |
| NA20540 | 0.133455 | 0.131439 | 0.132432 |
| NA20541 | 0.151899 | 0.18809 | 0.171351 |
| NA20542 | 0.152664 | 0.150963 | 0.151808 |
| NA20543 | 0.140155 | 0.15274 | 0.146309 |
| NA20544 | 0.138169 | 0.150927 | 0.144478 |
| NA20581 | 0.124474 | 0.129213 | 0.126899 |
| NA20582 | 0.148361 | 0.157051 | 0.152755 |
| NA20585 | 0.140719 | 0.152807 | 0.14664 |
| NA20586 | 0.167269 | 0.172444 | 0.169879 |
| NA20588 | 0.130268 | 0.15914 | 0.14387 |
| NA20589 | 0.16202 | 0.142857 | 0.152402 |
| NA20752 | 0.13982 | 0.171131 | 0.156213 |
| NA20754 | 0.138365 | 0.152257 | 0.145405 |
| NA20756 | 0.16285 | 0.157293 | 0.160121 |
| NA20757 | 0.136066 | 0.164782 | 0.150529 |
| NA20758 | 0.153589 | 0.180901 | 0.167739 |
| NA20759 | 0.146749 | 0.164441 | 0.155822 |
| NA20760 | 0.131555 | 0.175676 | 0.153085 |
| NA20761 | 0.156224 | 0.1554 | 0.155797 |
| NA20765 | 0.174579 | 0.192225 | 0.183673 |
| NA20766 | 0.14676 | 0.1595 | 0.153125 |
| NA20768 | 0.139837 | 0.142745 | 0.141317 |
| NA20769 | 0.127846 | 0.133438 | 0.130795 |
| NA20770 | 0.138549 | 0.171846 | 0.155452 |
| NA20771 | 0.164724 | 0.158228 | 0.161505 |
| NA20772 | 0.184902 | 0.211726 | 0.198587 |
| NA20773 | 0.143423 | 0.148058 | 0.145717 |
| NA20774 | 0.124444 | 0.127406 | 0.125903 |
| NA20778 | 0.112641 | 0.128405 | 0.120382 |
| NA20783 | 0.160247 | 0.161079 | 0.160674 |
| NA20785 | 0.18018 | 0.179688 | 0.179929 |
| NA20786 | 0.16735 | 0.175911 | 0.171705 |
| NA20787 | 0.131018 | 0.16679 | 0.149504 |
| NA20790 | 0.1424 | 0.145985 | 0.14418 |
| NA20792 | 0.156598 | 0.171946 | 0.164474 |
| NA20795 | 0.181748 | 0.178378 | 0.180069 |
| NA20796 | 0.174851 | 0.167217 | 0.170809 |
| NA20797 | 0.17843 | 0.187994 | 0.183221 |
| NA20798 | 0.116939 | 0.1251 | 0.121175 |
| NA20799 | 0.135425 | 0.168478 | 0.152461 |
| NA20800 | 0.14 | 0.131313 | 0.135678 |
| NA20801 | 0.169916 | 0.186441 | 0.178756 |
| NA20802 | 0.134771 | 0.148018 | 0.141459 |
| NA20803 | 0.175314 | 0.209318 | 0.193358 |
| NA20804 | 0.160273 | 0.166263 | 0.16335 |
| NA20805 | 0.099196 | 0.123457 | 0.111407 |
| NA20806 | 0.158954 | 0.18091 | 0.169393 |
| NA20807 | 0.162665 | 0.184727 | 0.174044 |
| NA20808 | 0.141447 | 0.142857 | 0.14218 |
| NA20809 | 0.11841 | 0.168186 | 0.143824 |
| NA20810 | 0.158177 | 0.183993 | 0.1716 |
